# Lipoprotein glomerulopathy associated with the Osaka/Kurashiki *APOE* variant: two cases identified in Latin America

**DOI:** 10.1186/s13000-021-01119-x

**Published:** 2021-07-26

**Authors:** Joaquim Nelito da Silveira-Neto, Guilherme Jinson de Oliveira Ahn, Precil Diego Miranda de Menezes Neves, Vinicius Augusto Ferreira Baptista, Stanley de Almeida Araújo, David Campos Wanderley, Andréia Watanabe, Elieser Hitoshi Watanabe, Neide Missae Murai, Eny Maria Goloni Bertollo, Osvaldo Merege Vieira-Neto, Márcio Dantas, Sergio Ricardo de Antônio, Roberto Silva Costa, Maria Alice Sperto Ferreira Baptista, Miguel Moysés-Neto, Luiz Fernando Onuchic

**Affiliations:** 1 Division of Nephrology, Hospital de Base, FAMERP/FUNFARME, São Jose do Rio Preto, Brazil; 2 Nephrology Service of Ribeirão Preto, Ribeirão Preto, Brazil; 3 Division of Nephrology, University of São Paulo School of Medicine, São Paulo, Brazil; 4 Division of Molecular Medicine, University of São Paulo School of Medicine, Avenida Doutor Arnaldo, 455 – Sala 4304, São Paulo, SP 01246-903 Brazil; 5 Catanduva Medical School, Catanduva, Brazil; 6 Division of Pathology, Federal University of Minas Gerais, Belo Horizonte, Brazil; 7 Nephropathology Institute, Belo Horizonte, Brazil; 8 Genetic Service, Hospital de Base, FAMERP/FUNFARME, São Jose do Rio Preto, Brazil; 9 Division of Nephrology, Ribeirão Preto School of Medicine - University of São Paulo, Ribeirão Preto, Brazil; 10 Division of Pathology, Ribeirão Preto School of Medicine - University of São Paulo, Ribeirão Preto, Brazil; 11 Division of Renal Pathology, Hospital de Base, FAMERP/FUNFARME, São Jose do Rio Preto, Brazil

**Keywords:** *APOE* gene, Apolipoprotein E, Case report, Lipoprotein, Molecular diagnosis, Kidney biopsy

## Abstract

**Background:**

Lipoprotein glomerulopathy (LPG) is a rare autosomal dominant disease caused by mutations in *APOE*, the gene which encodes apolipoprotein E. LPG mainly affects Asian individuals, however occasional cases have also been described in Americans and Europeans. Herein we report two unrelated Brazilian patients with LPG in whom genetic analyses revealed the APOE-Osaka/Kurashiki variant.

**Case presentation - case 1:**

A 29-year-old Caucasian male sought medical attention with complaints of face swelling and foamy urine for the last 3 months. He denied a family history of kidney disease, consanguinity, or Asian ancestry. His tests showed proteinuria of 12.5 g/24 h, hematuria, serum creatinine 0.94 mg/dL, albumin 2.3 g/dl, total cholesterol 284 mg/dL, LDL 200 mg/dL, triglycerides 175 mg/dL, and negative screening for secondary causes of glomerulopathy. A kidney biopsy revealed intraluminal, laminated deposits of hyaline material in glomerular capillaries consistent with lipoprotein thrombi. These findings were confirmed by electron microscopy, establishing the diagnosis of LPG. His apolipoprotein E serum level was 72 mg/dL and genetic analysis revealed the *APOE* pathogenic variant c.527G > C, p.Arg176Pro in heterozygosis, known as the Osaka/Kurashiki mutation and positioned nearby the LDL receptor binding site.

**Case 2:**

A 34-year-old Caucasian man sought medical assessment for renal dysfunction and hypertension. He reported intermittent episodes of lower-limb edema for 3 years and a family history of kidney disease, but denied Asian ancestry. Laboratorial tests showed BUN 99 mg/dL, creatinine 10.7 mg/dL, total cholesterol 155 mg/dL, LDL 79 mg/dL, triglycerides 277 mg/dL, albumin 3.1 g/dL, proteinuria 2.7 g/24 h, and negative screening for secondary causes of glomerulopathy. His kidney biopsy was consistent with advanced chronic nephropathy secondary to LPG. A genetic analysis also revealed the Osaka/Kurashiki variant. He was transplanted a year ago, displaying no signs of disease relapse.

**Conclusion:**

We report two unrelated cases of Brazilian patients with a diagnosis of lipoprotein glomerulopathy whose genetic assessment identified the *APOE*-Osaka/Kurashiki pathogenic variant, previously only described in eastern Asians. While this is the second report of LPG in Latin America, the identification of two unrelated cases by our medical team raises the possibility that LPG may be less rare in this part of the world than currently thought, and should definitely be considered when nephrotic syndrome is associated with suggestive kidney biopsy findings.

## Background

Lipoprotein glomerulopathy (LPG) is a rare autosomal dominant disease characterized by glomerular accumulation of lipoproteins as intracapillary thrombi [1]. It is more often reported in eastern Asian countries, however cases have also been observed in Europe and America [2–4]. LPG is caused by pathogenic variants in *APOE*, the gene which encodes apolipoprotein E (ApoE). This protein is a component of lipoproteins, including high-density lipoproteins (HDLs), very-low-density lipoproteins (VLDLs), intermediate-density lipoproteins (IDLs) and triglycerides [1, 5]. The *APOE* mutations most frequently associated with LPG are the Sendai [6] and Kyoto [7] variants (p.Pro163Arg and p.Arg43Cys, respectively), but other variants have been reported (Table 1) [8–10].

**Table 1:** Description of the *APOE* variants associated with lipoprotein glomerulopathy, including cDNA change (nucleotide), protein change (amino acid position in pre-apoE), variant identification and amino acid position in mature apoE, and position in the protein domain structure

cDNA change (nucleotide)	Protein change (amino acid change in the 317-residue pre-apoE)	Variant identification and amino acid change in the 299-residue mature apoE	Protein Domain
c.475_483del9	p.159_161delLRK	141_143del (Tokyo)	Heparan sulphate proteoglycan binding site
c.480_488del9	p.160_162delRKL	142_144del (Maebashi)	Heparan sulphate proteoglycan binding site
c.488G>C	p.R163P	R145P (Sendai)	Heparan sulphate proteoglycan binding site
c.494G>C	p.R165P	R147P (Chicago)	Heparan sulphate proteoglycan binding site
c.502C>G	p.R168G	R150G (Okayama)	LDL receptor binding site
c.502C>T	p.R168C	R150C (Modena)	LDL receptor binding site
c.503G>C	p.R168P	R150P (Guangzhou)	LDL receptor binding site
c.61G>T	p.R43C	R25C (Kyoto)	Other sites
c.394C>T	p.R132C	R114C (Tsukuba)	Other sites
c.509C>A	p.A170D	A152D (Las Vegas)	Other sites
c.518T > C	p.L173P	L155P (Chengdu)	Other sites
c.520_573del	p.174_191del	apoE1(156-173del)	Other sites
c.527G>C	p.R176P	R158P (Osaka/Kurashiki)	Other sites
c.644 C>G	p.S197C	S197C (Toyonaka)	Other sites
c.742G>T	p.D248Y	D230Y (Hong Kong)	Other sites

This report presents two cases of unrelated Brazilian patients with nephrotic syndrome whose investigation led to a diagnosis of LPG and identified the very rare Osaka/Kurashiki *APOE* variant as the disease etiology.

## Case presentation

### Case 1

A 29-year-old Caucasian male sought medical attention due to face swelling and foamy urine for the past 3 months. He denied a family history of kidney disease, consanguinity, or Asian ancestry, and reported hypothyroidism with continuous use of levothyroxine 150 μg qd. His physical examination revealed blood pressure within the normal range and 2+/4+ lower-limb edema.

Urinalysis showed proteinuria (4+/4+), dysmorphic hematuria (54/high power field) and 24-h proteinuria of 12.5 g. Serum laboratorial tests included creatinine 0.94 mg/dL, eGFR 109 mL/min/1.73m^2^ (CKD-EPI), total protein 3.6 g/dL, albumin 2.3 g/dL, cholesterol 284 mg/dL, LDL 200 mg/dL, HDL 49 mg/dL, and triglycerides 175 mg/dL. Blood cell counts, serum complement and hydroelectrolytic parameters were within the normal ranges. Hepatitis B and C and HIV serologies were negative as well as the screening for diabetes and auto-immune diseases. Serum and urine protein electrophoresis did not detect anomalous proteins and echography revealed kidneys with normal size and features.

**Fig. 1:**
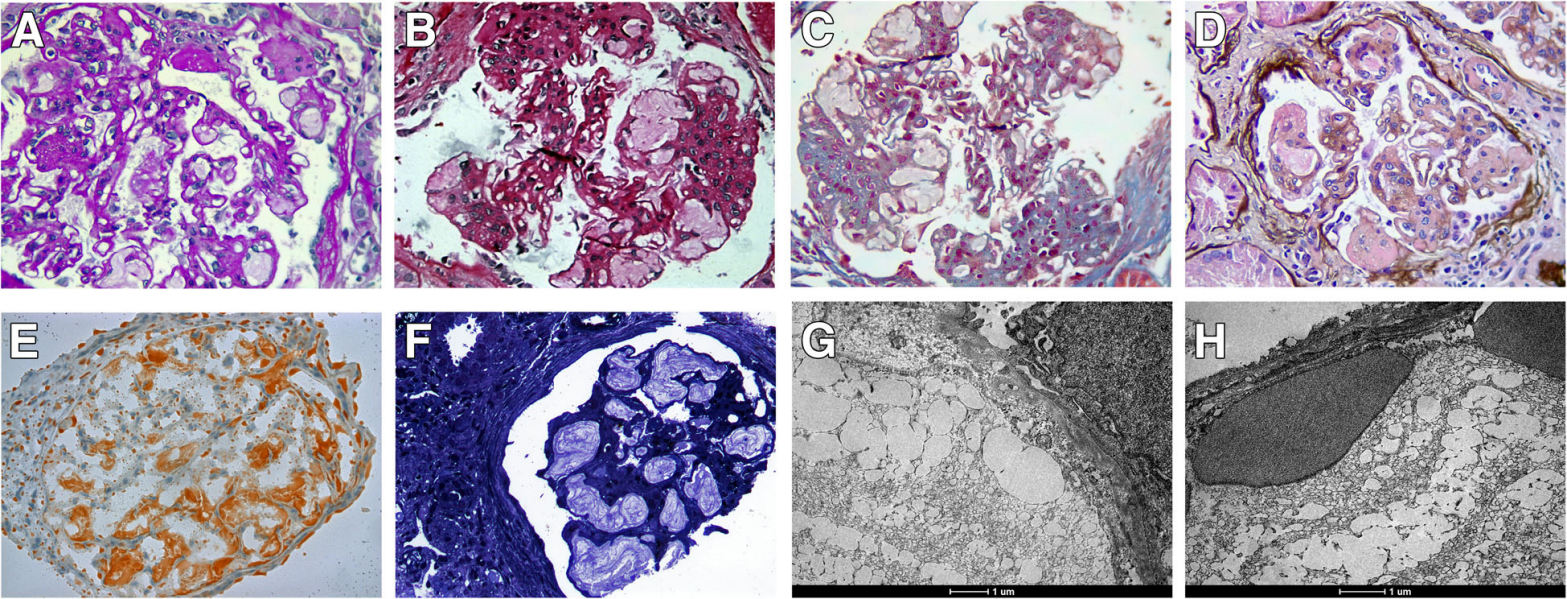
Kidney biopsy. Case 2. **A**, **B**, **C**, **D** Glomerulus with increased mesangial matrix and cellularity, displaying open capillary loops filled with amorphous, acellular, lipidic and pale material observed on PAS, Picrosirius, Masson’s trichrome and Jones silver methenamine staining, respectively; and **E** Oil Red staining showing lipidic material within the glomerular capillaries (400X). Case 1. **F** Toluidine blue staining revealing glomerulus with intense luminal deposition of lipidic material. Cases 2 (**G**) and 1 (**H**): Transmission electron microscopy obtained after re-inclusion of paraffin-embedded material in resin, stained with osmium tetroxide and ruthenium red, showing lipoprotein pseudo-thrombi constituted of vacuolized material inside the capillary loops (16.500X magnification)

The patient underwent a kidney biopsy (Fig. 1) with light microscopy evincing 12 glomeruli, two of them globally sclerosed. All glomeruli with open capillary loops showed increased volume and mild expansion of mesangial matrix, with slight hypercellularity. Intraluminal deposits of hyaline material were observed in glomerular capillaries with a compact appearance, pale and laminated, leading to loop distension consistent with lipoprotein thrombi. Basement membrane thickening was detected in some capillaries, while tubular atrophy and interstitial fibrosis were present in approximately 30% of the cortical area and a mild lymphocytic infiltrate was seen in fibrotic areas. Immunofluorescence was negative for immunoglobulins and complement fractions. Electron microscopy revealed dilated glomerular capillary loops with luminal obstruction by thrombi formed by acellular and transparent material associated with vesicle formation, a pattern consistent with lipoprotein thrombi. The diagnosis of LPG was therefore established.

**Fig. 2:**
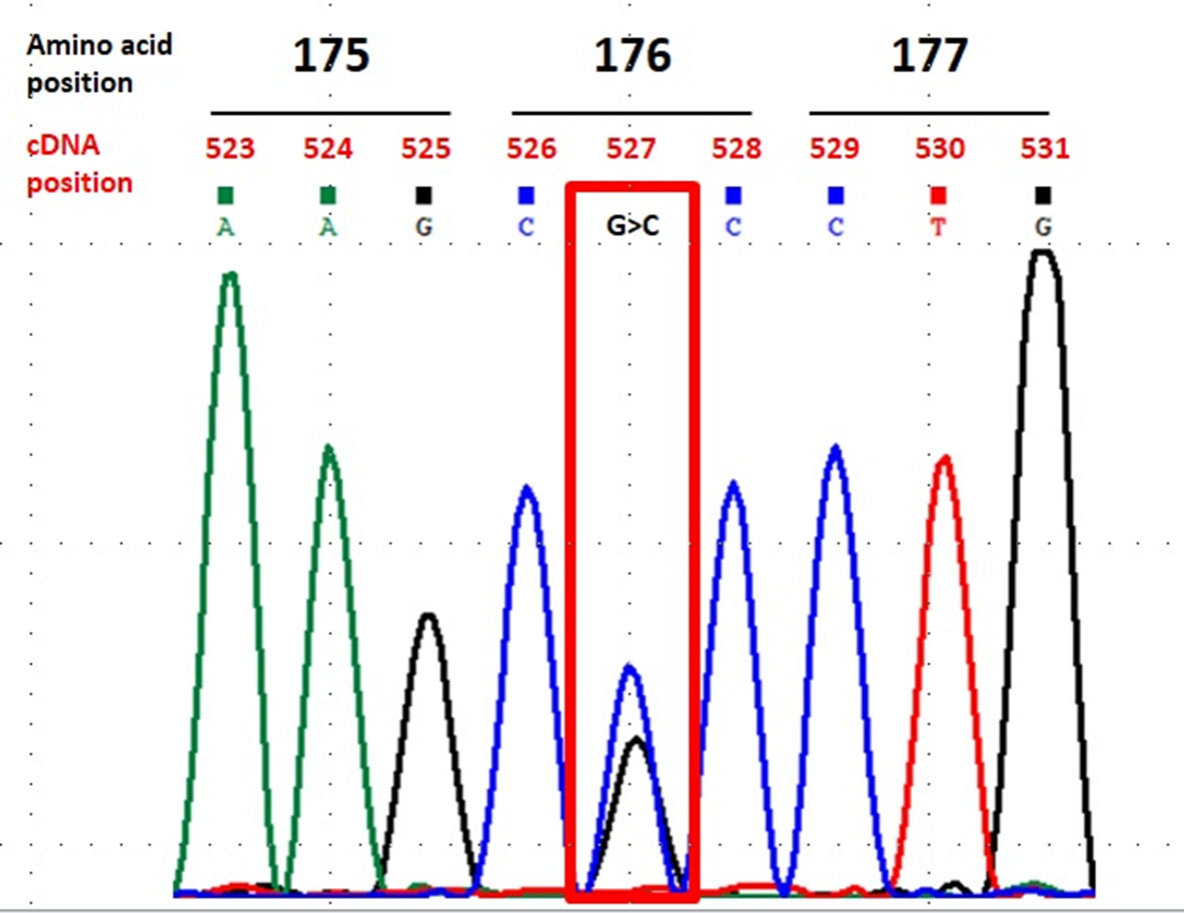
Patient 1’s chromatogram showing the c.527G > C variant in the *APOE* gene in heterozygosity, known as apoE-Osaka/Kurashiki variant, which leads to the p.Arg176Pro amino acid substitution

Serum apolipoprotein E was 7.2 mg/dL (normal range: 2.3–6.2 mg/dL). An investigation with next-generation sequencing applied to a gene panel directed to familial hypercholesterolemia identified the pathogenic variant c.527G > C, p.Arg176Pro in *APOE*, called Osaka/Kurashiki [8–11], in heterozygosity (Fig. 2). This variant is not described in the gnomAD, 1000 genomes or ABRAOM databases, and the corresponding amino acid (aa) position is conserved in mammals. This variant is classified as likely pathogenic according to the American College of Medical Genetics and Genomics criteria [12], reaching high pathogenicity scores on SIFT (0.01) and DANN (0.996), and a moderate score on Polyphen2 (0.89). The patient remains on simvastatin 20 mg qd and ezetimib 10 mg qd after 3 years, with serum creatinine of 2.37 mg/dL and 24-h proteinuria of 3.7 g.

### Case 2

A 34-year-old Caucasian man came to a nephrology service for renal dysfunction and systemic hypertension. The patient reported intermittent episodes of lower-limb edema for the last 3 years. He denied Asian ancestry, but referred a family history of renal disease, affecting his sister, aunt and cousin. His physical examination revealed hypertension, but an absence of lower-limb edema.

Laboratorial tests showed BUN 99 mg/dL, serum creatinine 10.7 mg/dL (CKD-EPI 5.6 mL/min/1.73m^2^), absence of electrolytic imbalances, metabolic acidosis (venous gasometry with pH 7.25 and bicarbonate 12 mEq/L), hemoglobin 6.2 g/dL, cholesterol 155 mg/dL, HDL 22 mg/dL, LDL 79 mg/dL, triglycerides 277 mg/dL, serum albumin 3.1 g/dL, and 24-h proteinuria 2.7 g. Serologies for hepatitis B and C and HIV and screening for auto-immune disorders and diabetes were negative. Serum and urine electrophoresis did not show abnormal protein peaks. Echographic analysis revealed a mild reduction in renal size and increased echogenicity.

A kidney biopsy showed 16 glomeruli, 14 of them globally sclerosed (Fig. 1). The two non-sclerosed glomeruli displayed endothelial cells with expanded volume obstructing the capillary lumen, cells with foamy cytoplasm, and segmental mesangial expansions. Interstitial fibrosis and tubular atrophy were diffuse and associated with hyaline arteriosclerosis. Sudan staining in a frozen specimen was positive in endothelial cells. Immunofluorescence showed C3 (+ 3/+ 3) and IgM (+ 2/+ 3) with segmental and global distribution following an entrapping pattern. Electron microscopy revealed dilated glomerular capillary loops with lipid droplets of variable sizes in the capillary lumen, a finding consistent with lipoprotein thrombi. These findings were consistent with advanced chronic nephropathy secondary to LPG. The patient progressed to renal replacement therapy after 2 months of conservative management. Whole exome sequencing also revealed the *APOE* c.527G > C, p.Arg176Pro variant in heterozygosity (Fig. 2). The patient was transplanted a year ago after 4 years on RRT.

## Discussion and conclusions

Human preapoliprotein E contains 317 aa, while the cleavage of an 18-residue signal peptide gives rise to its 299-aa mature form, assembled as a homotetramer. ApoE is mainly produced in the liver and plays a significant role in lipoprotein plasma clearance. *APOE* encodes three apoE isoforms, apoE2, apoE3 and apoE4, with apoE3 most often being present in the general population. The p.Arg176Pro substitution hits the repeat domain 5, located near the K175 succinylation site and the LDL receptor-binding site. Eight of the 15 variants associated to LPG involve the replacement of arginine with another residue, most of them located in the LDL receptor and heparan sulphate proteoglycan binding sites or close to them. Structural abnormalities in apoE reduce its capacity to bind LDL and IDLs, impairing catabolism and their clearance, while increase its binding affinity to other molecules such as heparin. As a result, *APOE* mutations can lead to specific glomerular diseases, including *APOE* homozygote glomerulopathy, membranous nephropathy-like apoE deposition disease and lipoprotein glomerulopathy [1]. *APOE* mutations have been identified in most LPG cases. It must be noted that morphological findings of intraluminal pale-staining and laminated lipid or lipoprotein thrombi are strongly consistent with APOE glomerulopathy, even if those findings are only present in one glomerulus. Such morphological findings were in fact present in both cases, raising a high suspicion for this glomerulopathy. The only differential diagnosis is fat emboli; however, the clinical presentation is completely distinct as LPG presents itself as a proteinuric disease (most often nephrotic syndrome) and fat emboli as an acute kidney injury.

Fifteen mutations have been described up until now. While apoE-Sendai and apoE-Kyoto particularly affect Japanese and Chinese individuals, apoE-Kyoto and other *APOE* pathogenic variants have been found elsewhere [4]. The first Latin American case was reported in 2014 with no genetic analysis [2], whereas a previous report identified the apoE-Chicago variant in a Mexican descendant [13]. The dysfunction increases lipoprotein plasma levels and leads to forming apolipoprotein and lipoprotein glomerular deposits. Asymptomatic carriers of *APOE* mutations have been identified, however, and not all mice harboring the apoE-Sendai variant developed LPG, suggesting that other factors may also play a role in the disease [9].

LPG usually manifests in adulthood and predominantly affects males. Hyperlipoproteinemia type III (increased LDL and triglyceride levels), high levels of plasma apolipoproteins, proteinuria and microhematuria are common findings, and nephrotic syndrome and progressive loss of renal function are often observed. The typical LPG histology comprises glomeruli with increased size due to ectasia of capillary loops, filled with light and lamellated eosinophilic thrombi. Electron microscopy confirms small lipid vacuoles typical of lipoproteins. Specific staining for fat or apoE can be performed in frozen tissue [1, 5].

Only five cases harboring the Osaka/Kurashaki variant have been described so far (Table 2) [8–11]. The first patient was a 26-year-old male with no family history of consanguinity, nephropathy, or dyslipidemia [8], with mild proteinuria, no hematuria, normal renal function, hypertriglyceridemia and increased VLDL. His kidney biopsy was positive for apoE antigen in the thrombi. The patient was treated with fibrate, reaching complete remission of proteinuria in 6 months. The second patient was a 45-year-old man with proteinuria, hypertriglyceridemia, hypercholesterolemia, normal renal function, proteinuria of 1-3 g/L, mild hematuria, an increased band between LDL and VLDL, and normal apoE serum levels. Treatment with probucol normalized his serum lipid levels [9]. Two other cases have recently been described in China. These patients were 22 and 28 year-old males, one of them with a positive family history of kidney disease [10]. The last case was just reported, but we do not have access to the corresponding information [11].

**Table 2:** Reported cases of Lipoprotein Glomerulopathy associated with the Osaka/Kurashiki *APOE* variant

Number of patients	Age at diagnosis	Sex	Family history	Country	Reference
1	26	Male	No	Japan	[8]
1	45	Male	Not available	Japan	[9]
2	22; 28	Male; Male	Yes; No	China	[10]
1	Not available	Not available	Not available	China	[11]
2	29; 34	Male; Male	No; Yes	Brazil	Our study

By analyzing the variants R176P, R163P and R165P, Georgiadou et al. [14] suggested that the substitution of arginine to proline in those positions induces a generalized unfolding of the N-terminal domain, including mild distortion in the protein spectral structure and significant loss of the helix conformation, essential for its binding properties. They also demonstrated that these structurally defective apoE are more sensitive to digestion by proteases and more prone to thermodynamic destabilization. These findings were not restricted to proline substitutions, but also to cysteine and aspartic acid in positions 43, 132 and 170 [15].

Current treatment of LPG includes hypolipemiants, in particular fibrates. Immunoadsorption with staphylococcal protein A and LDL apheresis using a heparin-induced extracorporeal lipoprotein precipitation system has also yielded positive results [1, 5]. The therapeutical roles of plasmapheresis, isolated LDL apheresis and inhibitors of Hidroxi-Methyl-Glutaril-CoA redutase are still controversial. As expected, LPG relapse in the graft has been reported in cases of kidney transplantation [16].

This is the first report of the apoE-Osaka/Kurashiki variant in non-eastern Asian patients with LPG and the third report of this disease in Latin Americans or descendants. Despite its rarity, the identification of two unrelated patients with this disease by our medical group raises the possibility that LPG may be less rare in Latin America than currently thought, and therefore should be considered in the differential diagnosis of nephrotic syndrome associated with suggestive findings in kidney biopsy.

## Data Availability

All meaningful data generated or analysed in this study are included in the manuscript.

## Abbreviations

ApoE: Apolipoprotein E
HDL: High-density lipoprotein
IDL: Intermediate-density lipoprotein
LPG: Lipoprotein glomerulopathy
VLDL: Very-low-density lipoprotein
